# A large post-stenting intramural hematoma in the left anterior descending artery caused by a small intimal calcium spur; should we respect the calcium shape?

**DOI:** 10.1186/s12872-023-03698-7

**Published:** 2024-01-06

**Authors:** Ahmad Samir

**Affiliations:** Faculty of Medicine, Cairo University, Cairo, Egypt

**Keywords:** Coronary calcification, Hostile morphology, Dissection, Intramural hematoma, Sub-stent

## Abstract

Coronary heavy calcification (HC) poses a sturdy challenge to percutaneous coronary intervention (PCI). Scores considering calcification length, thickness, or circumferential extent, are widely accepted to dictate upfront calcium modification to improve PCI outcomes. Although often marginalized, calcification shape (morphology) may require consideration during procedure planning in selected cases. This case demonstrates how a focal but spur-shaped calcification led to a massive proximal left anterior descending (LAD) dissecting intramural hematoma.

**Supplementary Information:**

The online version contains supplementary material available at 10.1186/s12872-023-03698-7.

## Introduction

Severe calcifications have been recognized as one of the most challenging complexities during percutaneous coronary interventions (PCI) [1]. Despite the introduction and advancement in several calcium modification techniques, coronary lesions with severe calcification still hold a higher risk of procedural failure, procedural complications, and higher risk of subsequent stent failure with poorer long-term outcomes [2, 3]. Angiography has limited sensitivity to appreciate and characterize coronary calcification, [4] thereby, intravascular imaging such as optical coherence tomography (OCT) or intravascular ultrasound (IVUS) has a significant impact on planning and technicalities particularly in complex calcified lesions [3].

## Case details

**Fig. 1:**
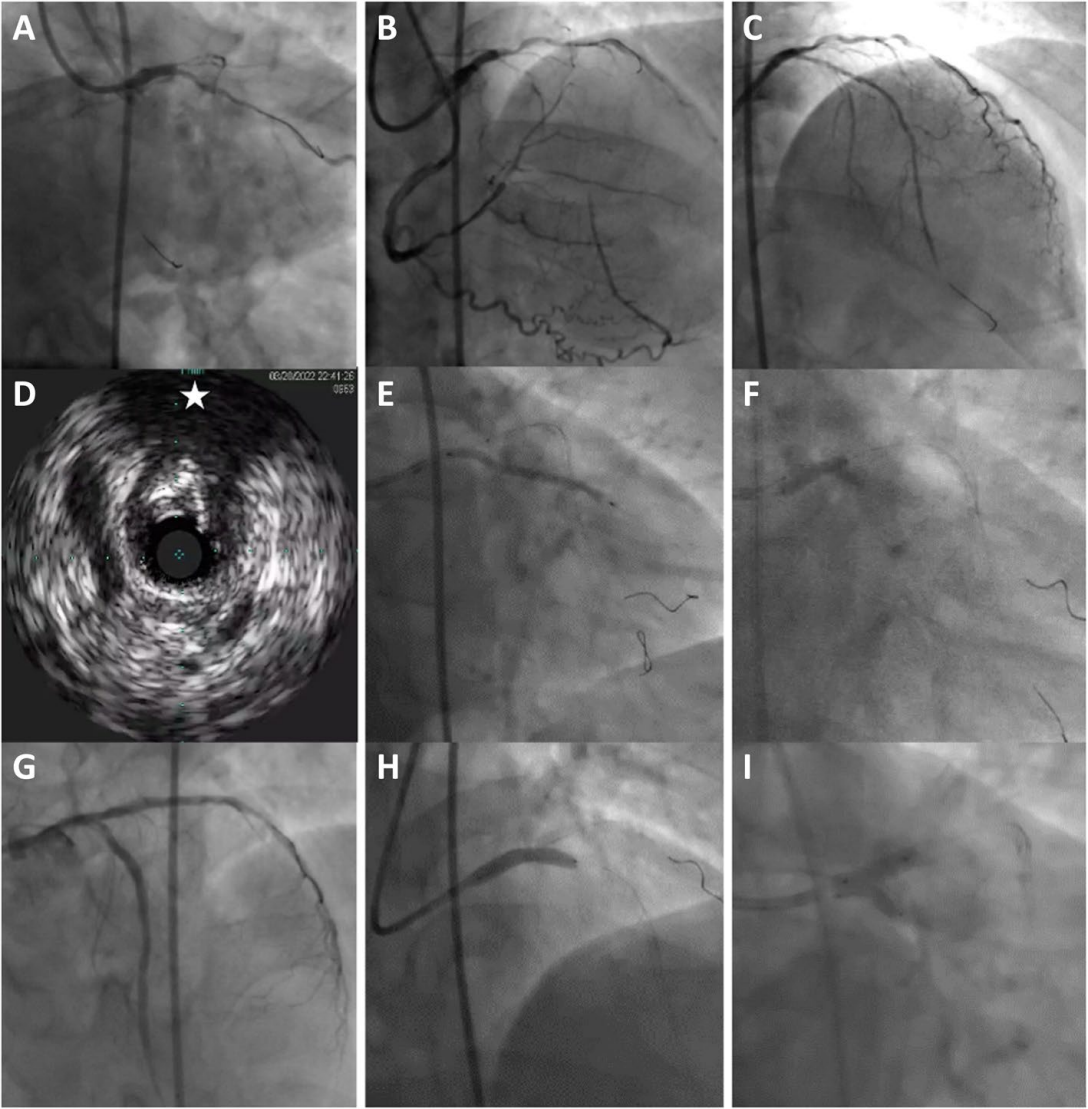
AP-caudal (**A**) and AP-cranial (**B**) views showing paraostial LAD CTO and severe LCx stenosis. After wiring and dilating the LAD CTO (**C**), IVUS revealed a pointed calcium spur (star) in proximal LAD (**D**). After stenting mid LAD, proceeded to DK-crush for the LM bifurcation with stenting distal LM-LCx while a balloon parking in LAD to crush (**E**). Afterwards, rewired the LCx and performed KBI (**F**). The proximal LAD showing a dissection flap (**G**), that was sealed by stenting LM-LAD (**H**), followed by rewiring the LCx and performing the second high pressure KBI (**I**). AP: antero-posterior; LAD: left anterior descending; LCx: left circumflex; IVUS: intravascular ultrasound; LM: left main; KBI: kissing balloon inflation

A 69-year-old male patient presenting with crescendo angina and found to have proximal left anterior descending (LAD) chronic total occlusion (CTO) and long severe stenosis in the left circumflex (LCx). For his comorbidities, heart team discussion voted for complex PCI to LAD CTO, long LCx lesion, planning to finish by left main (LM) DK-crush stenting. During the procedure, after wiring and dilatating the LAD CTO, IVUS revealed a spur-shaped calcification in proximal LAD. Being eccentric and focal, that calcified plaque was considered inconsequential and unlikely to hinder stents expansion or to require dedicated modification. After confirming adequate lesion expansion with 1:1 sized non-compliant balloons, proximal to mid LAD was stented first, planning to ensue with distal LM-to-LCx stent, then finalize with ostial LM-to-proximal LAD stent according to the standard technique. Proceeded to stent the distal LM-to-LCx, yet after the high-pressure kissing balloon inflation (KBI), we noticed a dissection in the native proximal LAD [opposite the calcium spur]. This called to expedite deploying the LM-to-LAD stent sealing the dissection, subsequently, wires were recrossed then performed the second and final KBI (Fig. 1).

**Fig. 2:**
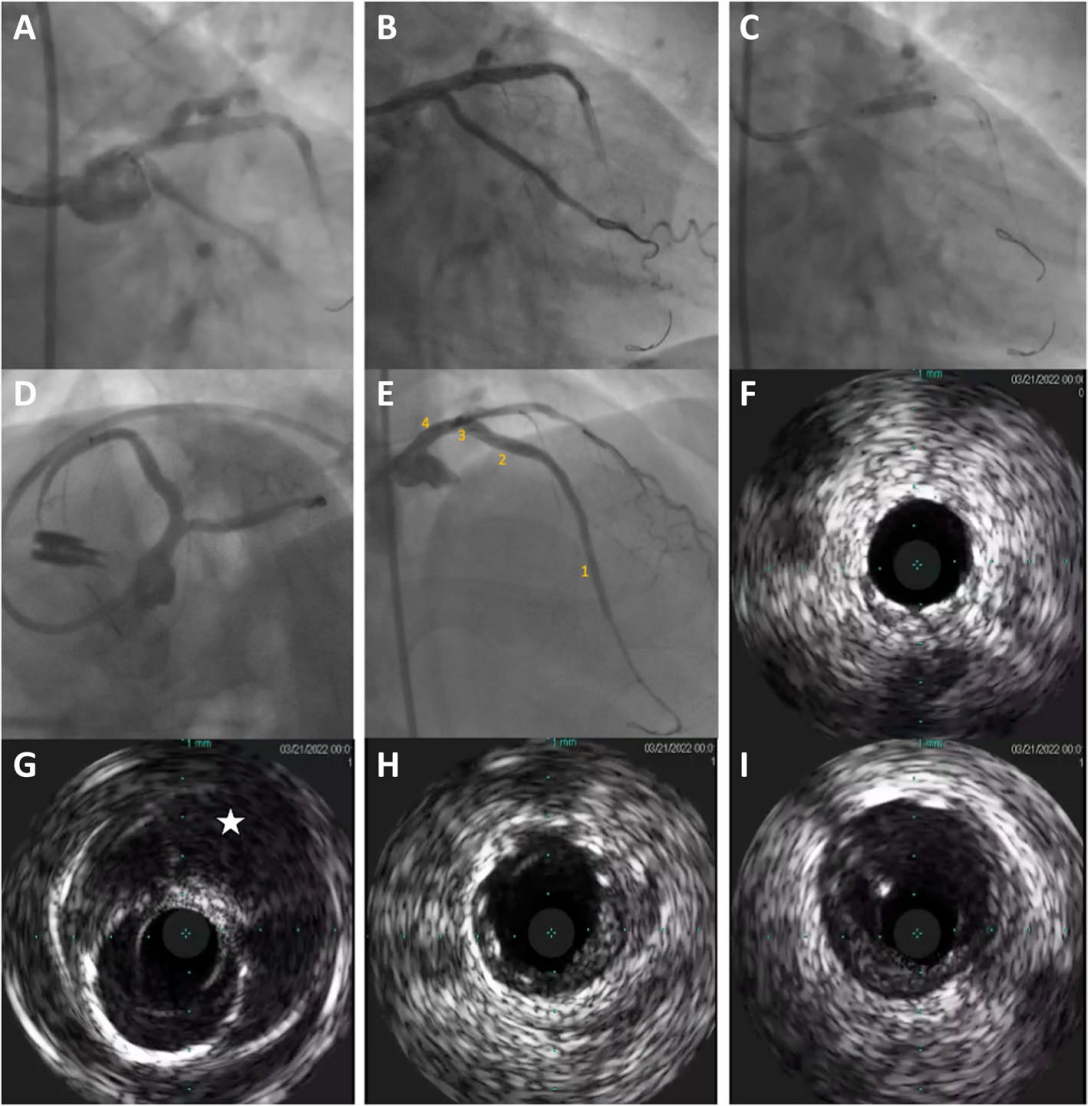
After post dilation, there was contrast extravasation from the proximal LAD (**A**), that remained refractory to prolonged balloon inflations (**B**), mandating to deploy a covered stent (**C**). After confirming adequate seal in caudal and cranial views (**D** and **E**), IVUS pull-back imaging was performed (**F**, **G**, **H** and **I** corresponding to 1, 2, 3 and 4 in (**E**) respectively), demonstrating that the extravasation was contained inside the LAD wall [sub-stent intramural hematoma (star), that likely had resulted from the stabbing by the calcific spur]. Abbreviations as Fig. 1

However, after the second high-pressure KBI, we appreciated contrast extravasation suggesting a perforation at proximal LAD [opposite the calcium spur]. Despite the angiographically significant contrast extravasation, surprisingly, the echocardiography revealed only a tiny rim of effusion not requiring pericardiocentesis. The sub-stent perforation was refractory to prolonged balloon occlusions, mandating deployment of a covered stent. After confirming angiographic seal with thrombolysis in myocardial infarction (TIMI) III flow in all branches, a final IVUS revealed that the extravasation was contained inside the LAD vascular wall forming a massive intramural hematoma surrounding the proximal LAD (Fig. 2).

## Discussion

Coronary calcification often complicates atherosclerotic plaques, and when dense and heavy, can hinder stent delivery and/or expansion [3]. Thereby, heavy calcification (HC) can significantly impair short- and long-term PCI outcomes [1]. In coronary chronic total occlusions (CTO), HC are more prevalent and complex, and often lead to lower success and more complications [5]. Pooled data from OCT and IVUS led to the development of scores evaluating the calcium length, thickness, and circumferential extent, to predict inadequate stents expansion, and thus to warrant upfront dedicated calcium modification techniques prior to stenting [6, 7]. However, calcium morphology is not systematically considered in these scores.

In this case, after successful wiring and dilatation of the LAD CTO, the IVUS revealed a sharply-pointed calcium spur in proximal LAD. Being eccentric, focal, and short, it was disregarded and considered inconsequential. However, throughout the procedure, whenever high pressure is exerted luminally in the proximal LAD [opposite the site of the calcific spur], a troubling complication occurs. The plausible explanation was the stabbing vascular-media injury occurring by the piercing of the calcific spur into the LAD wall during the first KBI (causing the dissection), then the second KBI (causing the dissecting intramural hematoma). Conceivably, because the vascular injury was limited to the tunica media, the massive sub-stent intramural hematoma was contained inside the vessel wall, hence, not resulting in complete vessel wall perforation or a tamponading effusion. Looking in retrospect, despite this calcium spur does not qualify the thresholds in the contemporary scores to warrant upfront calcium modification, [2, 6] yet its hostile morphology was significantly problematic. Probably, in selected cases, an antagonistic calcium morphology should be respected in procedural planning and should prompt consideration if ablation/modification is warranted.

## Conclusion

Although not among the contemporary criteria to consider modification, hostile calcification morphology can occasionally be problematic dictating special procedural considerations. Extreme caution and good preparation are necessary when treating antagonistic coronary calcifications.

### Supplementary Information

**Additional file 1.**

## Data Availability

Allowed upon reasonable request from the corresponding author.
