# Supplementary material for: A large post-stenting intramural hematoma in the left anterior descending artery caused by a small intimal calcium spur; should we respect the calcium shape?
Source: BMC Cardiovasc Disord. 2024 Jan 6;24:34. doi: 10.1186/s12872-023-03698-7 (PMC10771661; doi:10.1186/s12872-023-03698-7)
Supplement: Supplementary file 1 — Additional file 1. [file 12872_2023_3698_MOESM1_ESM.zip › Supplementary cine loops legend.docx]

Supplementary videos legends and links

| 1 | Baseline dual-injection in AP caudal view | https://drive.google.com/file/d/1UD-XnDrPw3231ckd7zX22BoJcdtTufQ6/view?usp=sharing |
| --- | --- | --- |
| 2 | Baseline dual-injection in AP cranial view | https://drive.google.com/file/d/1Tur-mLGC7IqNb37uHjkGNXlWhsdaCQtU/view?usp=sharing |
| 3 | After successful wiring and dilation of LAD CTO | https://drive.google.com/file/d/1UH0y4W4JsWNOYs678pyZeXmwYaP2Rxs2/view?usp=sharing |
| 4 | IVUS pull-back imaging revealing the focal calcium spur in proximal LAD not qualifying calcium modification | https://drive.google.com/file/d/1U7heywMahxk3JmraTWuWhhRXipl5r0Hq/view?usp=sharing |
| 5 | Stented mid LAD, then proceeded to DK-Crush to LM bifurcation, and performing the final kissing balloon inflation at high pressure | https://drive.google.com/file/d/1UF4sULhVIi3GSxMTBD__sGb2Cz3FYmN5/view?usp=sharing |
| 6 | Appreciating contrast extravasation from proximal LAD | https://drive.google.com/file/d/1U6r8W9B2F4hpqgzDVUVmwpTScSAno-uQ/view?usp=sharing |
| 7 | Advanced a balloon for prolonged balloon occlusion proximal to the perforation site | https://drive.google.com/file/d/1UOXMnDgFWNRlestpK17u-TMKTwuXoRGW/view?usp=sharing |
| 8 | Periodic transient deflate and check demonstrating that the extravasation was persistent | https://drive.google.com/file/d/1UDeeAAER0O9JH35sVVzI1RB8Xv2FItyB/view?usp=sharing |
| 9 | Proceeded to deploy a covered stent to seal the perforation that was resistant to prolonged balloon occlusion | https://drive.google.com/file/d/1UFGHftA_jdoWeWi-QClMVUVmm3IyQ51r/view?usp=sharing |
| 10 | IVUS imaging after the covered stent, demonstrating that the calcific spur caused dissecting injury in the tunica media of the LAD, causing extravasation into a massive intramural hematoma (inside the LAD wall) which explains why we did not find pericardial effusion/tamponade despite the excessive extravasation | https://drive.google.com/file/d/1TxGSKSyyA-O1Q8KrUyPUih1yB2tb09uL/view?usp=sharing |
| 11 | Final picture in AP caudal view | https://drive.google.com/file/d/1UIGkaLyWzfjyF4TfI_gDZcesvyT8gMXI/view?usp=sharing |
| 12 | Final picture in AP cranial view | https://drive.google.com/file/d/1U-yRYMvbWr4i_EilYMJIB32mFSTzOGH7/view?usp=sharing |
